# Bioinformatics Analysis Based on TCGA: MUC16 Mutation Correlates with Clinical Outcome in Gastric Cancer

**DOI:** 10.1155/2022/6734105

**Published:** 2022-08-23

**Authors:** Liang Huang, Shuang Zheng, Junhui Fu, Meng Zhang, Xiaogang Ge, Ning Mu

**Affiliations:** Department of General Surgery, Taizhou First People's Hospital, Taizhou, China

## Abstract

The prognosis of gastric cancer (GC) is difficult to predict due to the disease's complex genetic and phenotypic characteristics. MUC16 has been reported to be involved in the progression of several tumors. In this study, we aimed to explore whether MUC16 mutation had any impact on the prognosis or treatments of GC patients. Additionally, this analysis uncovered possible critical pathways related with these systems. On the cBioPortal, we were able to locate the pertinent data of patients with MUC16 mutations. And then, GSEA analysis identified differences in mRNA levels between mutant and wild-type MUC16 patients in terms of biological function annotation and pathways. The KEGG and GO analyses were also performed using the differentially expressed genes (DEGs). There were 139 individuals with GC who had the MUC16 mutation, which accounts for 32 percent, and the remaining patients had the MUC16 wild type. Survival assays revealed that patients with the MUC16 mutation had longer overall survival and disease-free survival. GSEA analysis revealed that cell cycle, cysteine and methionine metabolism, Huntington's disease, one carbon pool by folate, pyrimidine metabolism, pyruvate metabolism, RNA degradation, spliceosome, and valine leucine and isoleucine degradation were distinctly enriched in patients with MUC16 mutation type. Moreover, we identified 323 DEGs. Among them, 162 genes were upregulated, and 161 genes were downregulated. GO and KEGG assays indicated DEGs as enriched in pancreatic secretion, neuroactive ligand-receptor interaction, protein digestion and absorption, fat digestion and absorption, and glycerolipid metabolism. Overall, our data revealed that the MUC16 mutation in GC may affect the development of patients by altering several genes and pathways, indicating the importance of MUC16 mutation in the treatments of GC on an individual basis.

## 1. Introduction

Gastric cancer (GC) is the most common malignant tumor of the digestive system [1]. Despite the fact that there has been considerable advancement in terms of treatments, it remains the second leading cause of mortality due to cancer [2, 3]. In China, both the incidence and mortality rates of GC have been on the rise [4]. After surgery, patients typically undergo a variety of treatments, such as chemoradiation and chemotherapy, in an effort to delay or eliminate the likelihood of a cancer returning [5, 6]. Despite the fact that these treatments have led to an increase in patient survival rates, the overall survival rate for patients diagnosed with GC remains at roughly 30 percent worldwide [7, 8]. Therefore, the purpose of our research was to investigate potential biomarkers for the prognostic evaluation of GC patients.

Growing studies have confirmed that gene mutations are very important in disease program [9]. Chromosomal abnormalities, such as recurrent somatic mutations, copy number alterations, and oncogenic structural DNA rearrangements, have been uncovered in cases of primary germ cell cancer [10, 11]. In the field of ovarian cancer research, the biomarker MUC16 (which was formerly known as CA125) has been employed extensively, and its expression has been found to be related with the course of the disease [12]. Significant progress has been made in understanding the structure and activities of this protein, as well as the part it plays in essential processes, such as the prevention of epithelial damage and the development of human cancer [13, 14]. Ovarian, pancreatic, breast, and lung cancers have been found to exhibit aberrantly high levels of MUC16 [15–18]. MUC16 and its ligands have emerged as prospective therapeutic intervention targets thanks to their dysregulation and functional involvements. This has allowed monoclonal antibodies and immunotherapy to be utilized in their investigation. MUC16 is one of the genes in GC that is most susceptible to mutations. A variable MUC16 status may have an impact on the progression of cancer, the prognosis of the disease, and treatment options, and it may also cause certain patients to have a natural resistance or sensitivities to treatment tests [19, 20]. Thus, investigating the changes that take place in the important signaling pathways in patients who have the MUC16 mutation and determining the significance of these changes in tumor developments could assist us in gaining a deeper understanding of the pathogenesis of the disease, which will provide additional evidence for the individualized treatment of GC. In this research, we aimed to determine whether or not MUC16 mutations are linked to the clinical prognosis of GC patients.

## 2. Materials and Methods

### 2.1. RNA-Seq Data

From the database of the Cancer Genome Atlas (TCGA) (https://portal.gdc.cancer.gov/), an RNA-Seq dataset of GC was obtained, together with the clinical characteristics that corresponded to it. The website of the cBioPortal for Cancer Genomics was searched in order to collect the corresponding information associated to patients who had the MUC16 mutation (http://www.cbioportal.org/index.do).

### 2.2. Gene Set Enrichment Analysis (GSEA)

MUC16 mutation and wild-type patients were compared using GSEA v3.0 to find differences in gene mRNA expressions of biological functions, which allowed us to better comprehend the effects of the MUC16 mutation on various biological function gene sets in the GC patient population [21]. It was decided that there would be 5 different permutations. It was determined that the enrichment results were statistically significant if they met the criteria of a nominal *P* value threshold of 0.05 and a false discovery rate (FDR q-val) of less than 0.25.

### 2.3. Identification of Differentially Expressed Genes (DEGs)

An R package called EdgeR, which is used for analyzing abnormal expressions of RNA-Seq count data, was applied in accordance with the user's guide in order to screen DEG between patients with the MUC16 mutation and those with the wild-type form of GC. The following criteria were used to identify DEGs: |fold change (FC)| greater than 2; both the *P* value and the FDR were less than 0.05. The DEGs were applied for subsequent bioinformatics research.

### 2.4. Functional Enrichment Analysis

We used the “http://org.Hs.eg.db” package to convert gene symbols into Entrez IDs, and then, we used the “cluster Profiler,” “ggplot2,” and “enrich plot” packages to do pathway enrichment analysis on the DEGs based on the GO database and KEGG. The assays were performed on the DEGs. After applying the FDR approach, the *P* values were recalculated, and significantly enriched pathways were determined to have an FDR of 0.25 or lower.

### 2.5. Statistical Analysis

The Student's *t*-test was applied to compare the MUC16 expressions between the MUC16 mutation and wild-type GC tissues. Kaplan-Meier plots and log rank tests were used for survival analysis. Controlling the FDR in edgeR and GSEA required an adjustment for multiple testing using the Benjamini-Hochberg method, respectively. All statistical analyses were performed in RStudio (version 3.6.8), and we considered a *P* value <0.05 to be statistically significant.

## 3. Results

### 3.1. Data Information

**Figure 1:**
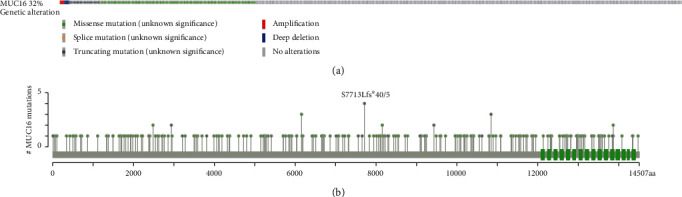
Mutation frequency (a) and types (b) of MUC16 in GC reproduced from the Cancer Genome Atlas (TCGA) datasets.

We collected the information for 441 GC specimens and nontumor specimens RNA-Seq datasets from the TCGA database. These datasets included complete follow-up data. There were 139 individuals with GC who had the MUC16 mutation, which accounts for 32 percent, and the remaining patients had the MUC16 wild type (Figure 1(a)). Amplification, truncating, and deep deletion mutations as well as inframe and missense variants spanned the entire gene. Other mutation categories included missense mutations (Figure 1(b)). The website known as cBioPortal for Cancer Genomics was applied in order to acquire these data.

### 3.2. Clinical Impact of MUC16 Mutation in GC Progress and Prognosis

**Figure 2:**
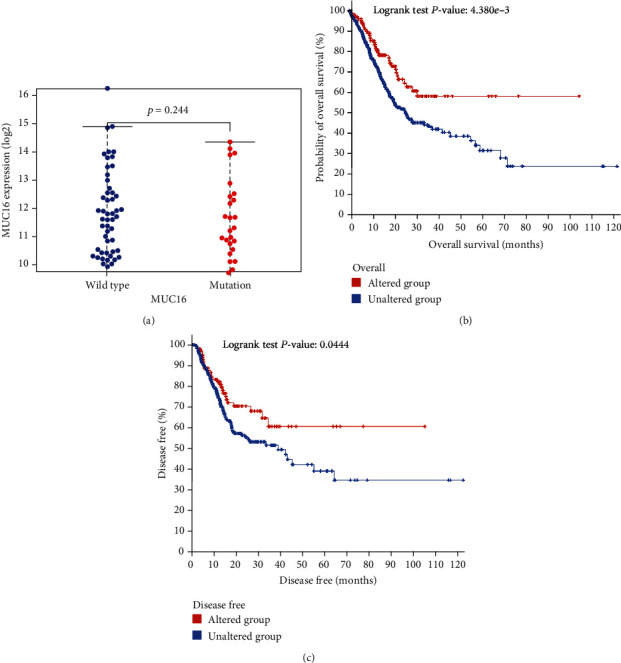
Mutations of MUC16 were related to the clinical outcome of GC patients. (a) The MUC16 mutation has been shown to correlate with the expression of mRNA. (b and c) The Kaplan-Meier survival curves, stratified by the MUC16 mutation, for patients with GC.

The second thing that we did was look into how the MUC16 mutation affected the progression of GC and the prognosis. We began by determining the levels of MUC16 in both the wild-type and mutant groups. According to the findings, MUC16 did not differ between GC specimens that had the wild-type and GC specimens that contained mutations (*P* > 0.244, Figure 2(a)). Survival assays revealed that patients with the MUC16 mutation had longer overall survival (Figure 2(b), *P* = 4.380e − 3) and disease-free survival (*P* = 0.0444, Figure 2(c)), suggesting that the MUC16 mutation suppressed the developments of GC. Patients who carried the MUC16 mutation can benefit from receiving intervention at an earlier stage.

### 3.3. GSEA

**Figure 3:**
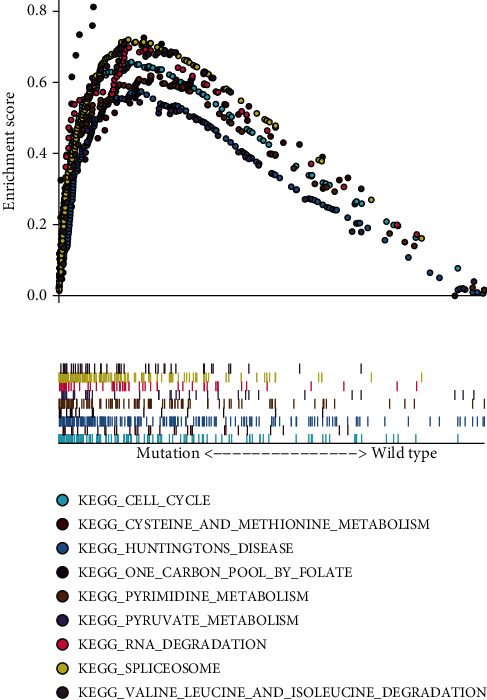
The gene set enrichment analysis (GSEA) was used to investigate the variations in gene enrichment observed in patients with the MUC16 wild-type and MUC16 mutant alleles.

All of these studies point to the fact that the MUC16 mutation played an important part in the progression of GC, the prognosis, and the choice of drugs. In order to study the mechanism and to collect more evidence, we began by analyzing the effects that the MUC16 mutation had on the processes that occur within cells. At initially, we used the GSEA method to conduct research on a number of different biological functional gene sets. As exhibited in Figure 3, we observe that cell cycle, cysteine and methionine metabolism, Huntington's disease, one carbon pool by folate, pyrimidine metabolism, pyruvate metabolism, RNA degradation, spliceosome and valine leucine, and isoleucine degradation were significantly enriched. Our findings revealed that the MUC16 mutation could suppress GC progression via regulating several tumor-related pathways in cell cycle, RNA degradation, and metabolism.

### 3.4. Identification of DEGs

**Figure 4:**
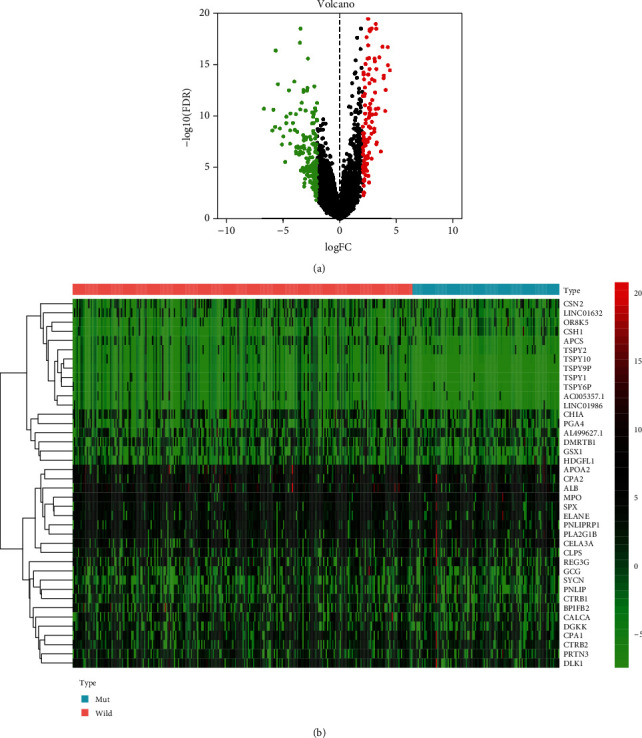
RNA-Seq datasets from 113 MUC16 mutation-bearing and other MUC16 wild-type GC patients were used for DEG screening. (a) Volcano plot of DEGs. (b) Heatmap of DEGs.

We identified the DEGs in order to conduct further research into the pathways and genes that were involved in the MUC16 mutation. For the purpose of DEG screen, RNA-Seq datasets from 113 GC patients carrying the MUC16 mutation and additional MUC16 wild-type patients were employed. As a result of the in-silico study, a total of 323 genes were determined to be DEGs by using the criterion of having a |fold change (FC)| more than 2.0 and *P* < 0.05. We found that 162 genes were upregulated, and 161 genes were downregulated throughout the entire set of genes mentioned above (Figure 4(a)). The heatmap of the DEGs is shown in Figure 4(b).

### 3.5. Functional and Pathway Enrichment Analyses of DEGs

**Figure 5:**
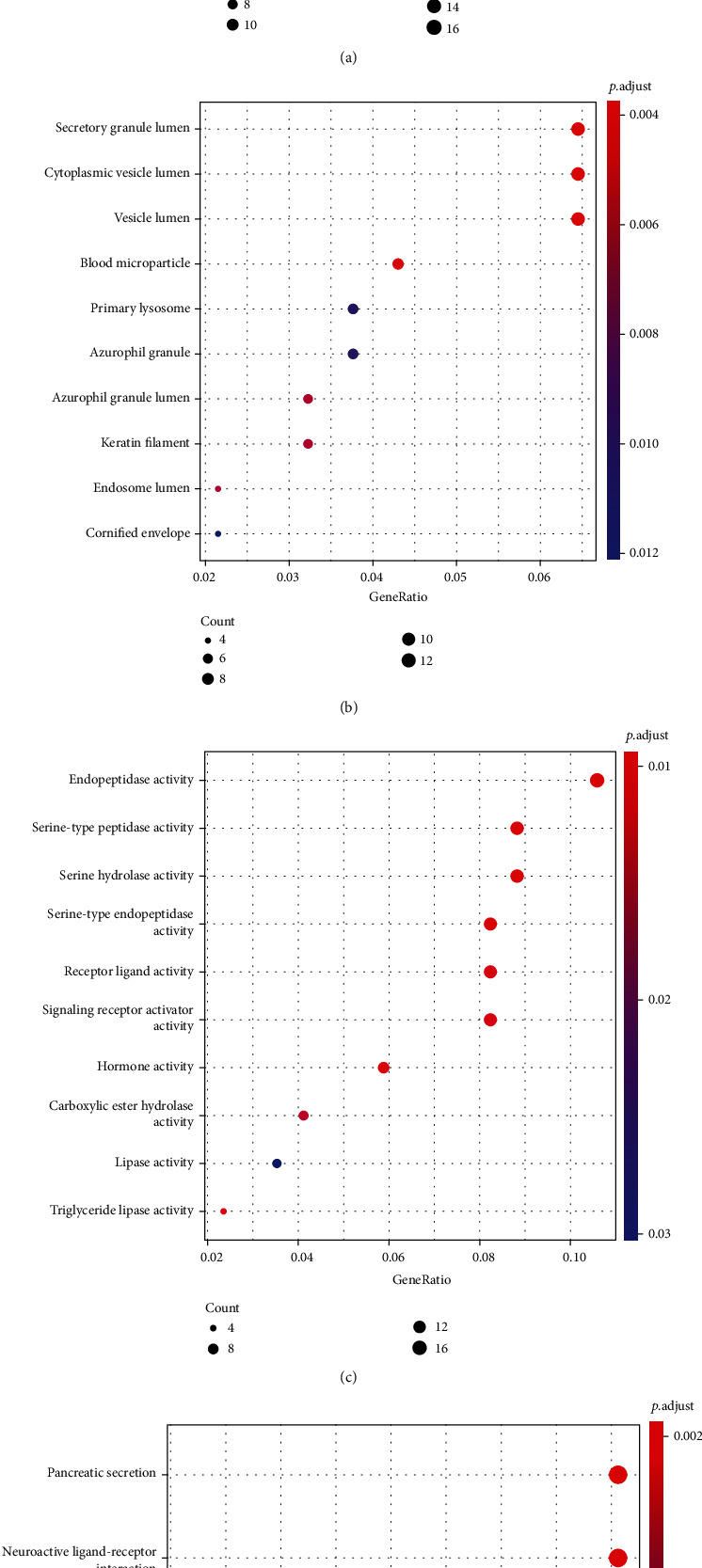
GO and KEGG assays were applied to explore the possible function of DEGs. (a–c) The GO enrichment terms of DEGs. (d) The KEGG assays of DEGs.

Enrichment studies for the GO and KEGG pathways were carried out in order to investigate the functional properties of the DEGs. In BP, the terms were mainly associated with epidermis development, keratinocyte differentiation, epidermal cell differentiation, skin development, and keratinization (Figure 5(a)). In CC, they were related to secretory granule lumen, cytoplasmic vesicle lumen, vesicle lumen, blood microparticle, and primary lysosome (Figure 5(b)). In MF, term enrichment mainly involved endopeptidase activity, serine-type peptidase activity, serine hydrolase activity, serine-type endopeptidase activity, and receptor ligand activity (Figure 5(c)). The results of KEGG assays revealed that the most distinctly enriched biological processes included pancreatic secretion, neuroactive ligand-receptor interaction, protein digestion and absorption, fat digestion and absorption, and glycerolipid metabolism (Figure 5(d)).

## 4. Discussion

The general outcome of advanced GC is quite poor, and only a few number of molecular targets have been demonstrated to be useful for GC [22, 23]. In order to successfully apply cancer precision medicine, the identification of biomarkers or signatures that can predict prognosis and therapeutic outcomes is an essential component. There have been reports of improvements in prognostic and therapeutic benefits brought about by the utilization of biomarkers in the treatment of colorectal cancer, breast cancer, lung cancer, and other types of cancer [24–26]. Nevertheless, the current initiatives consistently prioritize predictive accuracy over explanatory capacity. In recent years, an increasing number of studies have indicated that tumor mutation burden (TMB) is often referred to as a biomarker correlated with clinical responses to immune checkpoint blockade (ICB) in the treatment of nonsmall-cell lung cancer, cervical cancer, and GC [27–29]. Extensive sequencing of the genome using either next-generation sequencing (NGS) or whole-exome sequencing is used for TMB identification. Recently, the clinical prognosis of cancer patients can be predicted with the help of relatively straightforward procedures, such as the identification of single gene mutations, as has been demonstrated in a number of studies. Numerous studies have demonstrated that mutations in genes related to genomic integrity, such as TP53 and ATR, can lead to genomic instability and thus contribute to a high genomic mutation rate [30–32]. This is the case because these mutations can cause genomic instability. Therefore, investigating the association between the mutations of important genes and the clinical outcomes of GC patients is helpful for guiding immunotherapy on GC sufferers.

MUC16 is a unique glycoprotein, and its expression can be found in the cell membrane and a soluble form [13]. As a biomarker for ovarian cancer, MUC16 has been extensively used, and its expression has been shown to be related with the course of the disease [12]. Additionally, MUC16 has been shown to be useful in both the diagnosis and monitoring of the disease. In addition to this, it has been reported that increased quantities of soluble MUC16 were found in a variety of cancers, including breast cancer, mesothelioma, gastric cancer, colorectal adenocarcinoma, and some others [19, 33–35]. Comparative studies using MUC16 and other tumor markers, such as carcinoembryonic antigen, have been conducted on colorectal adenocarcinomas [36]. MUC16 has been found to have potential relevance as a future serological marker (CEA) [37]. A correlation has been shown between the upregulation of MUC16 and the progression of pancreatic ductal adenocarcinoma. According to the findings of a number of research, MUC16 may play a role in the evolution of GC. However, its association with clinical outcome was rarely reported. In order to encourage customized treatment, our goal was to determine the clinical importance of the MUC16 mutation in terms of the course and prognosis of GC. Our group further observed that about 32% of patients carried the MUC16 mutation among 441 cases, including missense mutation, splice mutation, truncating mutation, amplification, and deep deletion. According to the findings of a clinical study, GC patients with the MUC16 mutation have a significantly poorer prognosis in survival and disease-free survival. All of these findings suggest that the MUC16 mutation in GC patients should be given more attention in clinical practice, which is in line with the findings of some recent studies. The detection of mutations in tumor genes has been brought into clinical practice, which enables physicians to accurately determine the prognosis of patients and choose more effective and tailored treatment regimens. Our findings give evidence that individuals with gastric cancer who have the MUC16 wild type were more likely to develop distant metastases and that the clinical outcome of the disease is worse, suggesting that active treatments were essential for these patients in order to achieve a favorable prognosis. Patients with GC who have the MUC16 wild type may need more complete examinations to discover early metastatic tumor specimens. Alternatively, they may require earlier use of targeted therapies in order to cope with a poor illness prognosis.

Regarding the mechanisms, we performed an analysis on the RNA-Seq dataset of GC that was retrieved from TCGA in order to determine the important pathways and genes that are linked with the MUC16 mutation. These analyses were involved in the application of bioinformatics. GSEA analysis in the present study suggested that cell cycle, cysteine and methionine metabolism, Huntington's disease, one carbon pool by folate, pyrimidine metabolism, pyruvate metabolism, RNA degradation, spliceosome and valine leucine, and isoleucine degradation were significantly enriched. Our findings revealed that the MUC16 mutation may suppress GC progression via regulating several pathways in cell cycle, RNA degradation, and metabolism.

We identified the DEGs in order to conduct further research into the pathways and genes that were involved in the MUC16 mutation. There were a total of 323 genes that were found to be DEGs. GO assays revealed that 323 genes were involved in epidermis development, keratinocyte differentiation, epidermal cell differentiation, secretory granule lumen, cytoplasmic vesicle lumen, vesicle lumen, endopeptidase activity, and serine hydrolase activity. The results of KEGG assays revealed that the most distinctly enriched biological processes included pancreatic secretion, neuroactive ligand-receptor interaction, protein digestion and absorption, fat digestion and absorption, and glycerolipid metabolism. Our findings provided evidence that the MUC16 mutation plays a role in the progression of cells and sheds light on a potentially effective therapeutic target in patients who carry the MUC16 mutation, which can be used to form tailored treatment recommendations.

## 5. Conclusion

In conclusion, we demonstrated that the MUC16 mutation was related with the clinical outcomes of GC patients. Our research identified the probable pathways and key genes that were related to the MUC16 mutation in GC, which could potentially contribute to the development of therapeutic methods and predictive and prognostic tools for this particular subgroup of patients suffering from GC.

## Data Availability

The datasets used and/or analyzed during the current study are available from the corresponding author upon reasonable request.
